# Arts Therapies for Anxiety, Depression, and Quality of Life in Breast Cancer Patients: A Systematic Review and Meta-Analysis

**DOI:** 10.1155/2014/103297

**Published:** 2014-02-26

**Authors:** Katja Boehm, Holger Cramer, Thomas Staroszynski, Thomas Ostermann

**Affiliations:** ^1^Institute for Integrative Medicine, Faculty of Health, University of Witten/Herdecke, 58313 Herdecke, Germany; ^2^Department of Internal and Integrative Medicine, Kliniken Essen-Mitte, Faculty of Medicine, University of Duisburg-Essen, 45276 Essen, Germany; ^3^Hochschule für Kunsttherapie, University of Applied Sciences, 72622 Nürtingen, Germany

## Abstract

*Background*. Breast cancer is one of the most common types of cancer. However, only a few trials assess the effects of arts therapies. *Material and Methods*. We searched the Cochrane Central Register of Controlled Trials, PubMed, and Google Scholar from their start date to January 2012. We handsearched reference lists and contacted experts. All randomized controlled trials, quasi-randomized trials, and controlled clinical trials of art interventions in breast cancer patients were included. Data were extracted and risk of bias was assessed. Meta-analyses were performed using standardized mean differences. *Results*. Thirteen trials with a total of 606 patients were included. Arts therapies comprised music therapy interventions, various types of art therapy, and dance/movement therapies. The methodological quality ranged from poor to high quality with the majority scoring 3 of 4 points on the Jadad scale. Results suggest that arts therapies seem to positively affect patients' anxiety (standardized mean difference: −1.10; 95%, confidence interval: −1.40 to −0.80) but not depression or quality of life. No conclusion could be drawn regarding the effects of arts therapy on pain, functional assessment, coping, and mood states. *Discussion*. Our review indicates that arts interventions may have beneficial effects on anxiety in patients with breast cancer.

## 1. Introduction

### 1.1. Description of the Condition

Breast cancer is a condition whose diagnosis may result in extensive emotional, physical, and social suffering. It engenders stress and anxiety related to future prognosis and potential mortality. It may also cause uncertainty about changes in a woman's body image and treatment options. Patients may experience anxiety regarding surgical experience, coping with acute pain, treatment regimens, financial burdens of care, and disruptions of their personal and professional lives [1, 2].

Cancer patients are increasingly turning to complementary and alternative medicine (CAM) therapies to reduce symptoms, improve quality of life, and boost their ability to cope with stress. Different types of arts interventions have been used to alleviate symptoms and treatment adverse effects in women suffering from breast cancer. Arts therapies are made use of especially by motivated patients who want to actively participate in their healing process. Amongst others, from a large New Zealand health survey which sampled 12.529 people, aged 15 years and older, it is known that CAM users are more likely to be middle aged, rich, well educated, of European descent, and female [3]. They are more likely to have hardness to treat conditions and to be less well but actively try to maintain their health. In particular, when patients undergo acute treatment, arts therapies are recommended as well, when physicians detect the need for psychosocial support and therefore consult psychooncology services.

### 1.2. Description of the Interventions

Arts as therapy have become increasingly popular in a number of medical and health fields and the application ranges from working with children suffering from psychiatric disorders to elderly dementia patients. As the importance of psychosocial aspects of dealing with a cancer diagnosis and treatment has been better recognized and understood, the interest in arts therapies for breast cancer patients has also increased. Art therapy is an umbrella term for therapies such as dance and movement therapy, music therapy, and art therapy working with visual arts materials. The use of the artistic media as a means for therapy offers patients a way to communicate experiences, feelings, and needs, which are hard to express verbally. This possibility for an alternative way of communication can be important in particular for patients who are dealing with emotional conflicts and spiritual or existential issues. In reflecting on the image, music, or dance as well as on the process of its production with the arts therapist, one's resources can be activated or new ways of coping with the situation can be developed. At the same time the artistic process can be a way for experiencing one's own capability or to relax in times of straining physical treatment. Arts therapies therefore are increasingly used in psychooncology with the goal of psychosocial stabilization and support in the process of coping with the disease [4–6].

### 1.3. Other Research

Mainly, studies have looked at symptomatic effects such as anxiety and depression, two of the most commonly coexisting accompanying illnesses of breast cancer, while there are only a few empirical trials assessing the effects of art therapy on coping and quality of life of the breast cancer patient, apart from a number of case studies.

Wood et al. in 2011 carried out a systematic review of art therapy in adult cancer patients [7]. They concluded that art therapy is a psychotherapeutic approach that is being used to manage a spectrum of treatment-related symptoms and facilitate the process of psychological readjustment to the loss, change, and uncertainty characteristic of cancer survivorship. However, the review did not include a meta-analysis. Moreover, while breast cancer was the most prominent type of cancer in their review, Wood et al. did not include a separate analysis of studies that included only breast cancer patients [7]. Since patients with different types of cancer are heterogeneous in terms of sociodemographic factors, symptoms, treatment, and side effects, meta-analyses should focus on homogenous cancer groups [8]. Furthermore, the terminology is somewhat confusing since there is “art therapy” in which the term “art” refers to visual art and “arts therapies” as a main category for all forms also including therapies such as music therapy, dance therapy, and drama therapy.

Two Cochrane reviews were also published the same year—one investigating the effects of dance/movement therapy and the other of music therapy for improving psychological and physical outcomes in cancer patients [9, 10]. They found that one study suggested that dance/movement therapy may have a beneficial effect on quality of life. No effect of dance/movement therapy on body image was found. Furthermore, music interventions may have beneficial effects on anxiety, pain, mood, and quality of life in people with cancer and may have a small effect on heart rate, respiratory rate, and blood pressure. No review has yet assessed the available data on all forms of art therapy specifically in women with breast cancer.

### 1.4. Objective

It is the objective of this review to evaluate the current evidence and examine the effects of arts therapies (as defined above) on psychological outcomes in patients with breast cancer.

## 2. Material and Methods

PRISMA guidelines for systematic reviews and meta-analyses [11] and the recommendations of the Cochrane Collaboration [12] were followed.

### 2.1. Electronic Database Search

We searched the Cochrane Central Register of Controlled Trials (CENTRAL), PubMed, and Google Scholar. All databases were searched from their start date to January 2012. We handsearched reference lists and contacted experts. There were no language restrictions.

Search terms used for Cochrane Central Register of Controlled Trials were as follows: (breast or mamma) and (carcinoma or cancer) for breast cancer search and (art or dance, music or movement, or drawing or painting) for arts therapies search which were combined. These search terms were slightly modified for other databases.

### 2.2. Study Selection

We included all randomized controlled trials (RCTs), quasi-randomized trials, and controlled clinical trials of art interventions for improving psychological and physical outcomes in breast cancer patients. RCTs comparing the effect of art therapy with standard treatment or other treatments such as pharmacological medications in patients with breast cancer (aged >16 years) were eligible for inclusion. Trials that allowed other concomitant treatments were eligible, as long as they were given to both the art therapy and control groups. Participants undergoing biopsy and bone marrow transplantation were also included.

### 2.3. Outcome Assessment

Psychological outcome measures such as depression, anxiety, and quality of life as measured by validated instruments are the specific types of outcome measures that are closely being looked at in this review.

### 2.4. Data Extraction, Assessment of Methodological Quality, Allocation Concealment, and Risk of Bias Assessment

Data were extracted and risk of bias was assessed. One reviewer (KB) reviewed all searched articles to evaluate suitability for inclusion. If there was uncertainty, it was resolved by discussion with the corresponding reviewers (TS, TO, HC). After selection of studies, the aforementioned reviewer extracted data from the selected articles: author, year of publication, country of origin, study design, participants, outcome measures, arts therapy intervention, control intervention, main results, and adverse events. Data were extracted as intention-to-treat analyses; that is, all withdrawals and dropouts were assumed to be treatment failures, if trial reporting provided relevant information. Where possible, results were presented in meta-analyses using mean differences and standardized mean differences.

To assess the methodological quality of the respective studies, the Jadad score was adopted, which refers to randomization (0 to 2 points), blinding of the assessor (statistician, physician, assessor, or researcher, as cited in the original publications: 0 to 1 points), and dropout reporting (0 or 1 point) as indicators of methodological quality of a study [13]. Since double blinding was impossible, assessor blinding and therefore single blinding were rated. The total achievable score was 4 points. However, while it is clear what blinding of a “statistician” means, it is not very clear what blinding of “researcher” (as it was stated in some studies) may mean; therefore, it was assumed that this term referred to the outcome assessor.

Allocation concealment was assessed in accordance with the Cochrane guidelines [12]: (A) means adequate (telephone randomization or using consecutively numbered, sealed, opaque envelopes); (B) means uncertainty about the concealment (method of concealment is not known); (C) means inadequate (e.g., alternate days, odd/even date of birth, and hospital number).

Based on the methodological quality and the confidence in the results, the quality of evidence for each outcome was assessed according to the GRADE recommendations as high quality, moderate quality, low quality, or very low quality [14].

### 2.5. Data Synthesis and Statistical Analysis

Data were pooled using a fixed effects model. As crossover trials were included in the analysis, the generic inverse variance method was used. Studies were classified and combined in the analysis according to the outcome measure, intervention type, and/or control intervention. The impact of arts therapies on continuous outcomes at the end of treatment was expressed as standardized mean differences (SMD) with 95% confidence intervals (CI). Review Manager (RevMan) Version 5.1. Copenhagen: The Nordic Cochrane Centre, The Cochrane Collaboration, 2011 was used to generate forest plots of pooled SMDs with 95% CI. SMD was calculated as Hedge's *g* using a standardized Excel spreadsheet. For crossover trials, the calculation was adapted for intercorrelations between groups. Where no correlation was reported, it was estimated as 0.7 [15]. To address inconsistencies among the included studies, the *I* ^2^ test was used. The *I* ^2^ statistic indicates the proportion of variability across studies not explained by chance alone and the *I* ^2^ value of 50% or more was considered to be an indicator of substantial level of heterogeneity.

## 3. Results

### 3.1. Descriptive Statistics

**Figure 1:**
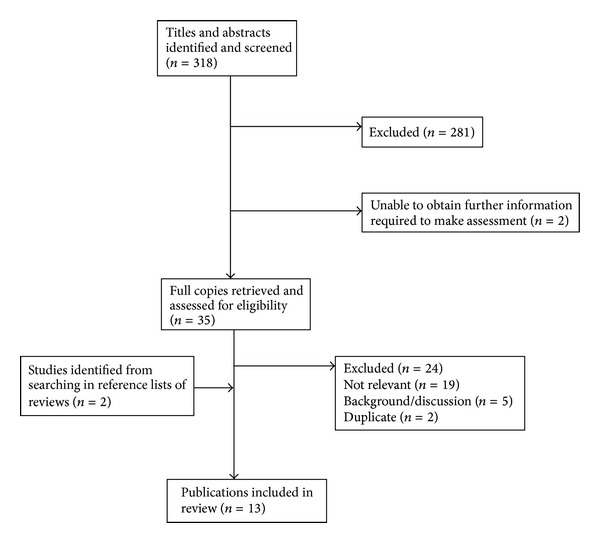
Flowchart of study selection process.

The literature search revealed 13 clinical controlled trials with a total of *n* = 606 breast cancer patients (Figure 1), [16–28] of which 2 were not randomized [17, 27].

One trial used a crossover design [17]. All studies were carried out between 2005 and 2011. The included studies employed therapies described as music therapy (*n* = 6), art therapy (*n* = 4), dance or movement therapy (*n* = 2), or music-assisted relaxation (*n* = 1). Most studies applied 2–6 sessions of art therapy to the breast cancer patients (range 2–66 sessions). Outcome measures included anxiety *n* = 4, QoL *n* = 5 (but only reported for *n* = 4), depression *n* = 5, mood states *n* = 3, symptoms/functional assessment *n* = 3, blood pressure *n* = 2, heart rate *n* = 2, social behavior/social desirability *n* = 2, body image *n* = 2, pain *n* = 1, coping *n* = 1, benefit finding *n* = 1, spiritual wellbeing *n* = 1, and shoulder range of motion *n* = 1. Number of participants in the treatment groups ranged between 10–54 patients and in the control group between 15–51 patients (Table 1).

### 3.2. Meta-Analysis

**Figure 2:**
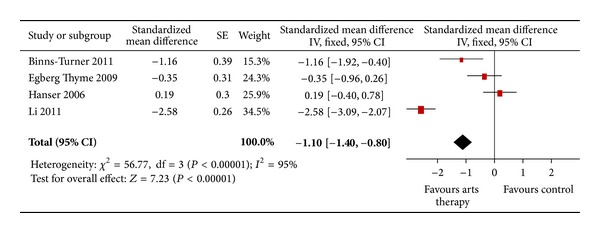
Forest plot of arts therapies for breast cancer, outcome parameter: anxiety.

For the outcome “anxiety” *n* = 4 trials were included and meta-analyzed. Results showed a mean difference of −1.10 [−1.40, −0.88]. This result is highly significant (*P* < 0.01) (see Figure 2).

**Figure 3:**
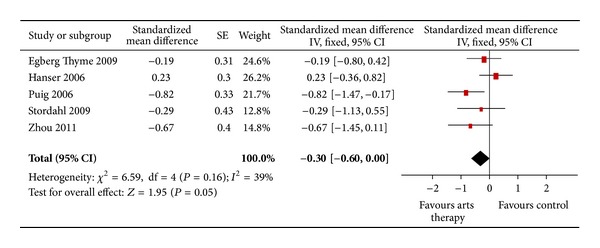
Forest plot of arts therapies for breast cancer, outcome parameter: depression.

For the outcome “depression” *n* = 5 trials were included and meta-analyzed. Results showed a mean difference of −0.30 [−0.60; 0.00] (*P* = 0.05). This difference is not significant (see Figure 3).

**Figure 4:**
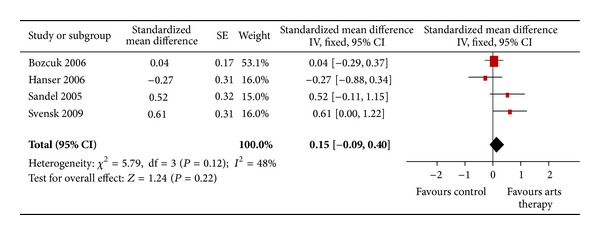
Forest plot of arts therapies for breast cancer, outcome parameter: quality of life.

For the outcome “quality of life” *n* = 4 trials were included and meta-analyzed. Results showed a mean difference of 0.15 [−0.09; 0.40] (*P* = 0.22) (see Figure 4).

### 3.3. Assessment of Methodological Quality

The methodological quality of included studies ranged from poor to high quality in the assessment with the majority scoring 3 [16, 23, 26, 28] or more points [20–22, 25] out of 4. One study received no [17], two studies one [19, 27], and two studies two points [18, 24] on the Jadad scale.

### 3.4. Quality of Evidence

Table 2 shows the assessment of quality of evidence for included studies according to the outcome. On a scale 1–9, anxiety and depression were rated to be symptoms of critical importance for breast cancer patients, whereas quality of life was rated as important. The quality of evidence for all outcomes was rated as medium meaning that the recommendation is as follows: in breast cancer patients the option of participation in arts therapies is being suggested and shown to be significantly effective for reduction of anxiety over control.

## 4. Discussion

This meta-analysis showed that arts therapies seem to positively affect the extent to which breast cancer patients score in anxiety and depression but not quality of life. It leads to the recommendation that in breast cancer patients the option of participation in arts therapies is being suggested and shown to be significantly effective for reduction of anxiety and depression over control.

Apart from case reports, there are currently only a small number of empirical studies investigating the effect of art therapy on psychological parameters such as coping and quality of life accompanying the breast cancer diagnosis and treatment.

### 4.1. Limitations

Not all studies included the same outcomes. There were a small number of studies per outcome. Therefore, the effectiveness of medical interventions for anxiety and depression could not be compared with those of arts therapies interventions. Three of the studies report on the same study population only different outcome measures [23, 26, 28].

### 4.2. Art Therapy Concepts

A qualitative study with *n* = 54 cancer patients who made use of art therapy during their outpatient experience whilst receiving chemotherapy was recently published [29]. Researchers did not only investigate the effects on quality of life or coping but they were also interested in identifying aspects of how patients experience art therapy as being “effective.”

During the analysis of the interviews, three dimensions of effectiveness became clear:

1. the process in general perceived as relaxing or stimulating due to the creative activity;
2. the therapeutic relationship allowing to communicate about oneself while feeling listened to;
3. the patient-image-art triadic relationship supporting the expression of emotions (in this case) through the painting and its symbolic function.

These findings correspond with the central categories describing processes of communication in art therapy as defined by Luzzatto: the direct communication between therapist and patient, a silent creative communication between patient and image, and the therapist trying to understand and elaborate the image together with the patient [30]. In this context one can conclude that the concept of art therapy in oncology is well suited. It makes it possible to identify and reflect on parallel-running processes of interactions within the individual, which are present on a number of different levels and constantly changing between physical and emotional state.

Nevertheless, the appropriate intervention in the wide spectrum from offering relaxation to the trying process of reflecting one's present situation of a possibly life-threatening illness needs to be chosen carefully. The often rapid changes in patients' physical and emotional state, especially in the acute treatment, require a high level of flexibility of the arts therapist. In order to support the process of coping with the disease, while not undercutting patient's individual strategies for holding up her mental stability, the arts therapist often needs to intervene in very different ways with each patient as well as sometimes in each session.

These coercively flexible strategies of intervention in combination with a broad range of concepts in arts therapies make it difficult to examine the effects of arts therapies. Accordingly, existing reviews and overviews of art therapy for cancer patients [7, 31] report about the complexity due to the very different therapy concepts that are being compared to assess the effect of art therapy in the context of the cancer disease and they also underline the necessity for further research projects, especially in regard to a further establishment of art therapy as an option to aid the care of cancer patients.

Since this review could not show a positive effect of art therapy on quality of life, what needs to follow is a comparative discussion of different intervention strategies according to phase of treatment in particular. In phases of great physical strain relaxation, oriented listening to music in receptive music therapy can result in fast effects, while those of a more confronting theme oriented intervention in visual art therapy possibly can be shown only in a longer interval.

Currently, a French team of researchers is enrolling patients in a phase 2 and phase 3 studies of art therapy for supportive care labeled “Impact of art therapy on fatigue and quality of life of patients treated with adjuvant radiotherapy for breast cancer” (trial identifier NCT01331629) [32].

Further studies investigate especially the influence of art therapy on symptoms of anxiety and depression. A significant improvement of their coping resources was found, for instance, in the RCT by Oster et al. for a group of breast cancer patients [23].

Overall, the option of participation in arts therapies can be recommended and has shown to be significantly effective for the reduction of anxiety over control in breast cancer patients.

## Figures and Tables

**Table 1:** Characteristics of the included studies.

First author	Year	Type of study	Type of arts therapy	Treatment duration	Type of control	Outcomes	Instruments	*N* treatment (as analysed)	*N* control (as analysed)
Binns-Turner	2011	RCT	Preoperational music	Pre-/intra-/postoperation (71–78 mins)	No music/usual care	Blood pressure, heart rate, anxiety, and pain	SAI, VAS	15	15

Bozcuk	2006	Crossover CCT	Music during chemotherapy	2 chemotherapy sessions	Session 1 served as control	QoL	EORTC QLQ C-30	18	18 (crossover)

Bulfone	2009	RCT	Music therapy	12 weeks, 15 mins each session	No music/usual care	Anxiety	STAI	30	30

Dibbell-Hope	2000	RCT	Dance, movement therapy	6 weeks, one session per week lasting 3 hrs each	Waiting list	Mood states, symptoms, body image, and social desirability	POMS, SCL-90-R, BWB, and MCSDS	15	16

Hanser	2006	RCT	Music therapy	3 for every 45 minutes	No music/usual care	QoL, Spiritual wellbeing, depression, heart rate, and blood pressure	FACT-G, FACIT-Sp HADS, and HADS	18	22

Zhou	2011	RCT	Music therapy	Hospital stay (13.6 ± 2 days) + 2 chemotherapy periods (18.9 ± 7.1 days), 2 daily for 30 mins each	No music/usual care	Depression	ZSDS	54	51

Li	2011	RCT	Music therapy	2 per day, 30 min session for maximum of 32.5 days	No music/usual care	Anxiety	SAI	54	51

Oester	2006	RCT	Art therapy	5 weeks total, 1 session per week	No art therapy/usual care	Coping	CRI	20	21

Puig	2006	RCT	Art therapies	4 sessions over 4 weeks, 60 mins each	Waiting list	Mood states	POMS	20	19

Sandel	2005	RCT	Dance, movement therapy	12 weeks: 6 weeks 2 sessions/week, 6 weeks 1 session, 1 hr each	Waiting list	Functional assessment, QoL, shoulder range of motion, and body image	FACT-B, ROM, BIS, and SF-36	19	16

Svensk	2009	RCT	Art therapy	60 min session/week for 5 weeks	No art therapy/usual care	Quality of life	EORTC, WHOQOL-BREF	20	21

Stordahl	2009	CCT	Music-assisted relaxation	4 sessions over 4 weeks, 60 mins each	Relaxation alone	Depression, mood states, and benefit finding	CES-D, POMS, BFS	10	10

Thyme	2009	RCT	Art therapy	5 weeks	No art therapy/usual care	Social behaviour, symptoms	SASB, SCL-90	20	21

Abbreviations: RCT: randomized controlled trial; CCT: controlled clinical trial; QoL: quality of life; SAI: Spielberger State Anxiety Scale; VAS: visual analog scale; EORTC QLQ C-30: quality of life of cancer patients; STAI: State Trait Anxiety Inventory; POMS: Profile of Mood States; SCL-90-R: Symptom Checklist 90 (Revised); BWB: Berscheid-Walster-Bohrnstedt Body Image Scale; MCSDS: Marlowe-Crowne Social Desirability Scale; FACT-G: Functional Assessment of Cancer Therapy—General; FACT-B: Functional Assessment of Cancer Therapy for patients with breast cancer; FACIT-Sp: Functional Assessment of Chronic Illness Therapy—Spiritual Well-being Scale; HADS: Hospital Anxiety and Depression Scale; ZSDS: Zung Self Rated Depression Scale; SAI: State Anxiety Inventory; CRI: Coping Response Inventory; ROM: range of motion; BIS: body image scale; SF-36: Short Form Health Survey; WHOQOL-BREF: World Health Organisation Quality of Life; CES-D: Center for Epidemiologic Studies Depression Scale; BFS: benefit finding scale; SASB: structural analysis of social behaviour, SCL-90: Symptom Checklist.

**Table 2:** GRADE assessment.

Quality assessment							Number of patients		Effect		Quality	Importance
Number of studies	Design	Risk of bias	Inconsistency	Indirectness	Imprecision	Other considerations	Art therapy	Control	Relative (95% CI)	Absolute		
Anxiety (follow-up 0–28 weeks, assessed with 3× STAI, 1× HADS-anxiety)												
4	randomized trials	no serious risk of bias	Some inconsistency	no serious indirectness	no serious imprecision	None	117	88	RR −7.09 (−8.32 to −5.87)	—	*⨁⨁* *⨁* MEDIUM	CRITICAL

Depression (follow-up 0–28 weeks, assessed with HADS, ZSDS, and CES-D)												
3	randomized trials	no serious risk of bias	Some inconsistency	no serious indirectness	no serious imprecision	None	117	88	RR −1.05 (−2.3 to 0.19)	—	*⨁⨁* *⨁* MEDIUM	CRITICAL

Quality of life (follow-up 0–28 weeks, assessed with 2× EORTC, 1× FACTG)												
3	randomized trials	no serious risk of bias	Some inconsistency	no serious indirectness	no serious imprecision	none	117	88	RR 1.89 (−4.77 to 8.56)	—	*⨁⨁* *⨁* MEDIUM	IMPORTANT

## References

[1] Poleshuck EL, Katz J, Andrus CH (2006). Risk factors for chronic pain following Breast Cancer surgery: a prospective study. *Journal of Pain*.

[2] Shelby RA, Taylor KL, Kerner JF, Coleman E, Blum D (2002). The role of community-based and philanthropic organizations in meeting cancer patient and caregiver needs. *Ca: A Cancer Journal for Clinicians*.

[3] Pledger MJ, Cumming J, Burnette M (2010). Health service use amongst users of complementary and alternative medicine. *New Zealand Medical Journal*.

[4] Borgmann E (2002). Art therapy with three women diagnosed with cancer. *Arts in Psychotherapy*.

[5] Hiltebrand EU, Malchiodi CA (1999). Coping with Cancer through Image Manipulation. *Medical Art Therapy with Adults*.

[6] Wood M, Waller D, Sibbett C (2005). Shoreline: the realities of working in cancer and palliative care. *Art Therapy and Cancer Care*.

[7] Wood MJM, Molassiotis A, Payne S (2011). What research evidence is there for the use of art therapy in the management of symptoms in adults with cancer? A systematic review. *Psycho-Oncology*.

[8] McNeely ML, Campbell KL, Rowe BH, Klassen TP, Mackey JR, Courneya KS (2006). Effects of exercise on breast cancer patients and survivors: a systematic review and meta-analysis. *Canadian Medical Association Journal*.

[9] Bradt J, Dileo C, Grocke D, Magill L (2011). Music interventions for improving psychological and physical outcomes in cancer patients. *Cochrane Database of Systematic Reviews*.

[10] Bradt J, Goodill SW, Dileo C (2011). Dance/movement therapy for improving psychological and physical outcomes in cancer patients. *Cochrane Database of Systematic Reviews*.

[11] Moher D, Liberati A, Teztlaff J, Altman DG (2009). Preferred reporting items for systematic reviews and meta-analyses: the PRISMA statement. *Annals of Internal Medicine*.

[12] Higgins JPT, Green S (2011). *Cochrane Handbook for Systematic Reviews of Interventions Version 5.1.0*.

[13] Jadad AR, Moore RA, Carroll D (1996). Assessing the quality of reports of randomized clinical trials: is blinding necessary?. *Controlled Clinical Trials*.

[14] Guyatt GH, Oxman AD, Vist GE (2008). GRADE: an emerging consensus on rating quality of evidence and strength of recommendations. *The British Medical Journal*.

[15] Cooper HM, Hedges LV, Valentine JC (2009). *The Handbook of Research Synthesis and Meta-Analysis*.

[16] Binns-Turner PG, Wilson LL, Pryor ER, Boyd GL, Prickett CA (2011). Perioperative music and its effects on anxiety, hemodynamics, and pain in women undergoing mastectomy. *AANA Journal*.

[17] Bozcuk H, Artac M, Kara A (2006). Does music exposure during chemotherapy improve quality of life in early breast cancer patients? A pilot study. *Medical Science Monitor*.

[18] Bulfone T, Quattrin R, Zanotti R, Regattin L, Brusaferro S (2009). Effectiveness of music therapy for anxiety reduction in women with breast cancer in chemotherapy treatment. *Holistic Nursing Practice*.

[19] Dibbell-Hope S (2000). The use of dance/movement therapy in psychological adaptation to breast cancer. *Arts in Psychotherapy*.

[20] Hanser SB, Bauer-Wu S, Kubicek L (2006). Effects of a music therapy intervention on quality of life and distress in women with metastatic breast cancer. *Journal of the Society for Integrative Oncology*.

[21] Zhou K-N, Li X-M, Yan H, Dang S-N, Wang D-L (2011). Effects of music therapy on depression and duration of hospital stay of breast cancer patients after radical mastectomy. *Chinese Medical Journal*.

[22] Li X-M, Zhou K-N, Yan H, Wang D-L, Zhang Y-P (2012). Effects of music therapy on anxiety of patients with breast cancer after radical mastectomy: a randomized clinical trial. *Journal of Advanced Nursing*.

[23] Oster I, Svensk A-C, Magnusson E (2006). Art therapy improves coping resources: a randomized, controlled study among women with breast cancer. *Palliative & Supportive Care*.

[24] Puig A, Lee SM, Goodwin L, Sherrard PAD (2006). The efficacy of creative arts therapies to enhance emotional expression, spirituality, and psychological well-being of newly diagnosed Stage I and Stage II breast cancer patients: a preliminary study. *Arts in Psychotherapy*.

[25] Sandel SL, Judge JO, Landry N, Faria L, Ouellette R, Majczak M (2005). Dance and movement program improves quality-of-life measures in breast cancer survivors. *Cancer Nursing*.

[26] Svensk A-C, Öster I, Thyme KE (2009). Art therapy improves experienced quality of life among women undergoing treatment for breast cancer: a randomized controlled study. *European Journal of Cancer Care*.

[27] Stordahl JJ *The influence of music on depression, affect, and benefit finding among women at the completion of treatment for Breast Cancer [open access dissertations]*.

[28] Thyme KE, Sundin EC, Wiberg B, Öster I, Åström S, Lindh J (2009). Individual brief art therapy can be helpful for women with breast cancer: a randomized controlled clinical study. *Palliative and Supportive Care*.

[29] Forzoni S, Perez M, Martignetti A, Crispino S (2010). Art therapy with cancer patients during chemotherapy sessions: an analysis of the patients’ perception of helpfulness. *Palliative and Supportive Care*.

[30] Luzzatto P, Holland JC (2010). Art Therapy. *Psycho-Oncology*.

[31] Geue K, Goetze H, Buttstaedt M, Kleinert E, Richter D, Singer S (2010). An overview of art therapy interventions for cancer patients and the results of research. *Complementary Therapies in Medicine*.

[32] http://clinicaltrials.gov/ct2/show/NCT01331629.

